# Lower Plasma Ghrelin Levels are Found in Women with Diabetes-Complicated Pregnancies

**DOI:** 10.4274/jcrpe.2504

**Published:** 2016-12-01

**Authors:** Rita Angélica Gómez-Díaz, Monica P. Gómez-Medina, Eleazar Ramírez-Soriano, Lucio López-Robles, Carlos A. Aguilar-Salinas, Renata Saucedo, Arturo Zarate, Adan Valladares-Salgado, Niels H. Wacher

**Affiliations:** 1 National Medical Center “Siglo XXI”, Mexican Social Security Institute, UMAE Hospital of Specialties, Unit of Medical Research in Clinical Epidemiology, Mexico City, Mexico; 2 UMAE Hospital of Specialties, Clinic of Obstetrics Gynecology, Mexico City, Mexico; 3 National Medical Center “La Raza”, Hospital of Gynecology Pediatrics 3A, Mexico City, Mexico; 4 National Institute of Medical Sciences and Nutrition, Department of Endocrinology and Metabolism, Mexico City, Mexico; 5 National Medical Center “Siglo XXI”, Mexican Social Security Institute, UMAE Hospital of Specialties, Unit of Medical Research in Endocrine Diseases, Mexico City, Mexico; 6 National Medical Center “Siglo XXI”, Mexican Social Security Institute, UMAE Hospital of Specialties, Unit of Biochemistry, Mexico City, Mexico

**Keywords:** Proinsulin, ghrelin, Diabetes, Hyperglycemia, neonates

## Abstract

**Objective:**

To evaluate the associations of glycemic control and gestational age with ghrelin and proinsulin levels in cord blood and mothers’ peripheral blood during pregnancy.

**Methods:**

This is a cross-sectional comparative study of twenty-four pregnant women with gestational diabetes (GD), 18 with type 2 diabetes mellitus (T2DM), and 36 without diabetes, as well as their neonates. Levels of proinsulin, ghrelin, and glycated hemoglobin A1c (HbA1c) were measured from maternal blood during the last week before caesarian delivery and in neonatal umbilical cord blood samples.

**Results:**

Mothers with GD and T2DM had significantly lower ghrelin levels compared to the healthy mothers (p<0.001). Maternal proinsulin was lower in women with GD than in women without diabetes (p<0.001). Proinsulin was significantly elevated in the neonates of women with GD and in women with HbA1c ≥6.5% (p<0.001). However, maternal ghrelin levels were higher (p=0.031) and neonate proinsulin levels lower in the pre-term offspring of mothers with GD (p=0.033). There was a negative correlation between HbA1c levels and birth weight (r=–0.407, p<0.001).

**Conclusion:**

Ghrelin levels were lower in pregnant women with diabetes, although pre-term birth appeared to reverse this trend in GD. Proinsulin levels were also low in pregnant women with diabetes and even lower in pre-term vs. at-term births. Both ghrelin and proinsulin levels were lower in pregnant women with diabetes and HbA1c of <6.5%. Thus, ghrelin participates in the adaptation to the caloric imbalance of diabetic pregnancy and may play a similar role in pregnancy-related complications, since high ghrelin concentrations may be necessary for normal fetal development.

**WHAT IS ALREADY KNOWN ON THIS TOPIC?**

Although ghrelin and proinsulin can regulate several metabolic pathways, few studies have evaluated these hormones in mothers with diabetes and their neonates.

**WHAT THIS STUDY ADDS?**

Our results indicate that pregnant women with gestational or type 2 diabetes had significantly lower ghrelin levels, compared to non-diabetic pregnant women. However, pregnant women with gestational diabetes had significantly lower proinsulin levels, compared to non-diabetic pregnant women. Thus, ghrelin participates in the adaptation to the caloric imbalance of diabetic pregnancy and may play a similar role in pregnancy-related complications, since high ghrelin concentrations may be necessary for normal fetal development.

## INTRODUCTION

Ghrelin helps modulate appetite and regulate several metabolic pathways. This peptide hormone is mainly produced in the stomach, although small amounts are also produced in the hypothalamus, kidney, heart, pancreatic cells, and placenta (1). Ghrelin has a role in both endocrine (e.g., the release of prolactin and adrenocorticotrophic hormone) and non-endocrine (e.g., stimulating gastric acid secretion and intestinal motility) functions (2,3). Ghrelin has two molecular forms: acylated ghrelin (acylated at the serine-3 residue) and des-acyl ghrelin. Women with gestational diabetes (GD) have lower levels of acylated ghrelin, which may reflect the inhibitory effect of insulin on ghrelin secretion (4). Although the role of ghrelin in fetal adaptation to intrauterine malnutrition is incompletely understood, there are studies which indicate a strong correlation between acylated and total ghrelin levels as a parameter for neonatal ghrelin regulation (5,6,7). Nevertheless, high ghrelin concentrations appear to be necessary for normal fetal development and this requires an optimal uterine environment that is free from hyperglycemia (8).

The relationships between ghrelin levels and various anthropometric and biochemical measurements remain controversial (9). Diabetes may also affect ghrelin concentrations, as plasma ghrelin levels are thought to decrease during hyperglycemia and hyperinsulinemia, although studies of ghrelin levels in pregnant women with diabetes are scarce (10).

Proinsulin is synthesized by the early embryo, before the differentiation of the pancreas. Proinsulin stimulates cardiogenesis and prevents apoptosis during neurulation (11). As sugars easily cross the placenta, the fetal pancreas responds to hyperglycemia by increasing insulin production. This process leads to fetal hyperinsulinemia, which affects carbohydrate, protein, and fat metabolism and is associated with an increased risk of metabolic diseases in adulthood (10,12). Furthermore, similar to insulin and insulin-like growth factor 1, proinsulin has an impact as a growth factor and this property may be associated with the higher incidence of congenital defects among children of diabetic mothers (11,13,14,15,16). Hyperglycemia can also induce metabolic damage by causing beta cell injury (17). Although long-term follow-up studies on this issue are lacking, alterations in maternal metabolism are reported to be associated with pancreatic islet hyperplasia, changes which may have long-term consequences for the fetus (18).

In this present study, using glycated hemoglobin A1c (HbA1c) levels, we evaluated the association of glycemic control with ghrelin and proinsulin concentrations in umbilical cord blood and maternal peripheral blood. We also evaluated the associations between maternal and neonatal ghrelin and proinsulin levels in preterm and term deliveries. Our hypothesis was that pregnant women with GD or type 2 diabetes mellitus (T2DM) would be more likely to have neonates with decreased ghrelin and increased proinsulin concentrations compared to the women without diabetes, which might be a risk factor for pre-term delivery.

## METHODS

This cross-sectional comparative study evaluated women with type 2 or GD and their offspring according to the American Diabetes Association criteria (19). We included 78 pregnant women and their neonates in the study. Of the women, 42 (53.8%) were diabetics. A group consisting of 36 (46.2%) healthy women and their offspring served as controls. The study and control groups were recruited from among consecutive pregnant women who were covered by our social security system and who had attended scheduled visits at the Hospital of Gynecology Pediatrics 3A (UMAE, National Medical Center “La Raza”) over an 11-month period. The exclusion criteria for mothers were type 1 diabetes mellitus (T1DM), pregnancy complications (e.g. pre-eclampsia), arterial hypertension, chronic renal or hepatic disease, cardiac failure, arrhythmia, cardiomyopathy, receiving steroids within 24 h after delivery, and serious maternal or fetal complications during the birth process. Also excluded were neonates born vaginally, those with an Apgar score <6 at 1 minute, with a short umbilical cord (unable to take blood sample), with sepsis, or with meconium in the amniotic fluid. The study was approved by the Ethics and Research Committee of the Mexican Social Security Institute and complied with the tenets of the Declaration of Helsinki. All participants provided written informed consent.

A blood sample was obtained from each mother during the last week before caesarian delivery and after a 12-hour fast and was used to measure plasma HbA1c, ghrelin, and proinsulin concentrations. Immediately after birth, a 10-mL cord blood sample was obtained for determination of total ghrelin and proinsulin concentrations. After the neonate became stable, supine body length (in millimeters, taken on a hard horizontal surface from crown to heel), unclothed weight (in grams), and cephalic perimeter (in centimeters) were measured. Gestational age was determined using the Capurro evaluation system (20). Infant weight was classified as adequate-, low-, or high-for-gestational-age according to the Lubchenco tables (21).

Whole blood HbA1c levels were determined using ion exchange high-performance liquid chromatography (normal range: 4-6%). The untreated samples were stored in aliquots at -80 °C until analysis. Total ghrelin and proinsulin concentrations were determined via radioimmunoassay using reagents from Millipore Corporation (MA, USA). The total ghrelin test has a sensitivity of 93 pg/mL, with intra- and inter-assay coefficients of variation (CVs) of 8.0% and 9.5%, respectively. The proinsulin test has a sensitivity of 2 pMol/L (normal fasting range: 7.9±1.5 pMol/L) and CVs of 5.0% and 10.1%, respectively.

### Definitions

Maternal HbA1c levels of ≥6.0% are generally considered indicative of inadequate glycemic control, according to the American Diabetes Association recommendations (19), while levels <6.5% are considered adequate (22). Therefore, we defined maternal hyperglycemia as a plasma HbA1c level of ≥6.5%. Any infant born before the completion of 37 weeks of gestation was classified as pre-term and those born at 37-42 weeks as a term infant. The infants were defined as small-for-gestational-age if they were below the 10th percentile for body weight, appropriate-for-gestational-age if between the 10^th^ and 90^th^ percentiles, and large-for-gestational-age if above the 90^th^ percentile in weight (21).

### Statistical Analysis

Analysis of variance or the Kruskal-Wallis test was used to compare anthropometric values and ghrelin, proinsulin, and HbA1c levels. A Pearson correlation coefficient test was used for data with a normal distribution and Spearman correlation coefficient for data with a non-normal distribution. Student’s t-test was used to compare parametric variables and the Mann-Whitney U test was used to compare the non-parametric variables, proinsulin levels in neonates of mothers with or without diabetes, proinsulin levels in pre-term and at-term neonates, and according to metabolic control. All analyses were performed using Statistical Package for the Social Sciences software (version 15; SPSS Inc., Chicago, IL, USA). The “General Lineal Models” module was used in lineal regression to adjust for the confounding factors of body mass index (BMI), age, disease duration, and HbA1c at the end of pregnancy. A p-value of <0.05 was considered significant.

## RESULTS

Table 1 shows the characteristics of the mothers and neonates. Among the mothers with diabetes, 57.1% (24/42) had GD and 42.9% (18/42) had T2DM. The durations of type 2 and GD were 29.8±23.4 months (range: 4-84 months) and 3.4±2.1 months (range: 1-8 months), respectively. Among women with diabetes, 54.8% (23/42) had an HbA1c value of <6.5%, 19% (8/42) were of normal weight, 50% (21/42) were overweight, and 31% (13/42) were obese. Dietary management was prescribed for 52.4% (22/42) of these women, while insulin therapy was necessary for 37.5% (n=9) of women with GD and for 61.1% of women with T2DM (n=11). Only 8.3% (3/36) of the non-diabetic mothers were overweight. It should be noted that one of the women without diabetes had an HbA1c of 6.2%. Nevertheless, both her fasting glucose (95 mg/dL) and insulin (12.7 μU/mL) levels were within normal ranges, and in the follow-up, she was not diagnosed as a diabetic in view of the principles suggested by Metzger et al (22).

Of infants born to mothers with type 2 or GD, 64.3% (27/42) were male and 45% (19/42) were pre-term. Of these neonates, 4.7% (2/42) were of low-for-gestational-age birthweight, 69% (29/42) of normal birthweight, and 26.3% (11/42) of high-for-gestational age birthweight. All neonates from non-diabetic mothers were born at term with normal birthweights and 55.6% (20/36) were male.

There was a significant difference in proinsulin levels between neonates who were born to mothers with or without diabetes (p<0.001) as well as between neonates from mothers with an HbA1c level of <6.5% or ≥6.5% (p<0.001) (Table 1).

Pregnant women with gestational or T2DM had significantly lower plasma ghrelin levels compared to women without diabetes (p<0.001). This difference remained significant after adjusting for BMI, age, disease duration, and HbA1c levels at the end of pregnancy. Maternal ghrelin concentrations were significantly higher in women without diabetes vs. women with diabetes (p=0.013). Ghrelin levels were also high in women with HbA1c levels <6.5% (vs. HbA1c of ≥6.5%) (p=0.01) (Table 2).

While maternal ghrelin levels were lower in the presence of diabetes, when only women with diabetes were evaluated, maternal ghrelin concentrations were higher in women who had pre-term deliveries (vs. term), and especially so in women with GD (p=0.031). The same trend was observed for women with T2DM, although the differences were not statistically significant (Table 3). There was no difference in proinsulin levels between women with and without diabetes. Pregnant women with T2DM and at-term birth had significantly higher proinsulin concentrations vs. pre-term birth (p=0.027), although this trend was not significant in women with GD (p=0.63).There was no difference in ghrelin concentrations between neonates who were born to mothers with or without diabetes. Furthermore, ghrelin levels were not modified by a maternal HbA1c level ≥6.5% or by pre-term birth. Nevertheless, negative correlations were observed between HbA1c concentration and birth weight (r=–0.407, p<0.001), ghrelin concentrations and birth weight among term neonates (r=–0.270, p=0.039), and between maternal ghrelin and neonatal ghrelin levels (r=–0.328, p=0.034).

Neonates born to mothers with diabetes had significantly higher proinsulin levels, regardless of glycemic control [adequate glycemic control (p<0.001) and inadequate glycemic control (p=0.026)]. Elevated proinsulin levels were observed in neonates born to women with T2DM and with an HbA1c value of <6.5% (177.6 pMol/L) and in neonates born to women with GD and an HbA1c value of ≥6.5% (80.9 pMol/L) (data not shown).

## DISCUSSION

The findings of this study support the concept that ghrelin affects the adaptive response to caloric imbalance. In this context, diabetic pregnancy can involve a positive or negative caloric balance, although women with T2DM typically have a caloric surplus. Our data show that women with gestational or T2DM had significantly lower plasma ghrelin concentrations at term compared to the non-diabetic controls. However, this difference was not observed for women with pre-term neonates, which may indicate that this is an obstetric complication that is caused by ambient stress and/or caloric deficiency. This interaction may explain the partial correction of low ghrelin plasma concentrations in women with pre-term birth compared to women with diabetes. Our results also suggest that ghrelin participates in the adaptation to the caloric imbalance of diabetic pregnancy and may play a similar role in pregnancy-related complications. Few reports have evaluated plasma ghrelin concentrations in women with diabetes and their offspring, although Kos et al (23) found lower plasma ghrelin levels at the end of pregnancy in women with T1DM. However, this finding was not replicated by Hehir et al (24), and Lappas et al (25) reported lower plasma ghrelin concentrations in women with GD, with persistence of this abnormality at 12 weeks post-partum predicting incident maternal diabetes. Aydin et al (26) found transitory low ghrelin levels in women with GD, although the levels normalized at 2 weeks post-partum. The same trend was observed in pregnant women with pre-gestational T2DM, although their ghrelin concentrations remained low compared to the control group. Our results extend the available evidence and indicate that maternal ghrelin concentrations decrease during pregnancy in women with type 2 or GD.

The low ghrelin concentrations during diabetic pregnancy may be related to maternal or placental factors. Insulin resistance is a common feature of T2DM that is exacerbated during pregnancy and is usually associated with decreased ghrelin secretion (27). The placenta also plays an important role in maintaining the appropriate circulating levels of maternal ghrelin during the later gestational stages. Therefore, diabetic pregnancy is a cause of endothelial dysfunction and premature placental aging, which may result in abnormal placental ghrelin secretion.

Our observation that pre-term birth partially reverses low ghrelin concentrations in pregnant women with diabetes is relevant, as maternal ghrelin concentrations do not vary significantly during a normal pregnancy (28). Interestingly, high ghrelin concentrations have been detected in the cord blood of pre-term and small-for-gestational-age infants (5,7). One study evaluated children of women with T1DM and reported that cord blood ghrelin concentrations negatively correlated with birth weight and that female infants had higher ghrelin concentrations. We also observed that weight-for-gestational-age negatively correlated with serum ghrelin in at-term neonates, which is similar to a previous report of weight-for-gestational-age being negatively correlated with neonatal serum ghrelin levels (29). However, another study reported that ghrelin levels did not differ between pre-term and at-term neonates (30). Nevertheless, maternal ghrelin at the end of a pregnancy is not correlated with fetal birth weight or placental weight (23), although there are no data regarding maternal serum ghrelin concentrations in pre-term neonates. These data suggest that maternal ghrelin may help control fetal growth, and ghrelin may be needed for fetal adaptation to abnormal uterine conditions, such as hyperglycemia (31). Additionally, abnormal ghrelin levels in the newborn may have long-term consequences in the regulation of appetite and weight (32).

Reports have consistently indicated that diabetes in pregnant women increases neonatal proinsulin concentrations, regardless of birth weight. For example, the fetal pancreas would respond to maternal hyperglycemia by increasing insulin production and subsequently cause beta cell hyperplasia in the islets of Langerhans (33,34). Although the effect of maternal diabetes on the conversion of proinsulin to insulin in the fetus is not known, our findings confirm reports which indicate that proinsulin levels are higher in neonates born to diabetic mothers (35). Proinsulin levels may also be a risk marker for the development of diabetes, metabolic syndrome, arterial hypertension, dyslipidemia, and other metabolic diseases (36).

The present study has several limitations. First, the sample size was small, so the conclusions may not be definitive. Secondly, approximately half of the mothers were receiving insulin, and this treatment heterogeneity may be a confounding factor. We also did not have the means, in this paper, to evaluate the differences in age and BMI of the subjects, which might be considered a confounding factor, as suggested by Tschop et al (37). However, both age and BMI were considered in the logistic regression. Also, we did not collect data regarding acylated ghrelin (the active form), although both acylated and unacylated ghrelin levels are altered by diabetes (38,39). Finally, we cannot compare ghrelin levels with those in other studies, due to the heterogeneity of measurement methodology.

In conclusion, ghrelin levels were lower in pregnant women with diabetes, although pre-term birth appeared to reverse this trend in GD. The proinsulin concentrations in pregnant women with diabetes were generally low, and this was particularly true among pregnant women with T2DM and pre-term birth (vs. at-term birth). Finally, among pregnant women with diabetes, proinsulin and ghrelin concentrations were lower in women with HbA1c of <6.5%. These data appear to indicate that ghrelin and proinsulin concentrations in pregnant women and their offspring depend on the type of maternal diabetes, gestational age at birth, and the degree of maternal glycemic control.

## Acknowledgments

The authors thank Ms. Susan Drier for helping prepare this manuscript, Leonardo Cruz-Reynoso and Luisa Sánchez-García for patient recruitment, Ms. Sandra Campos for taking the blood samples, and Mr. Ricardo Cesar Saldaña-Espinoza for helping with the statistical analysis.

## Ethics

Ethics Committee Approval: The study was approved by the Ethics and Research Committee of the Mexican Social Security Institute, complied with the tenets of the Declaration of Helsinki, Informed Consent: All participants provided written informed consent.

Peer-review: Externally peer-reviewed.

## Figures and Tables

**Table 1:**
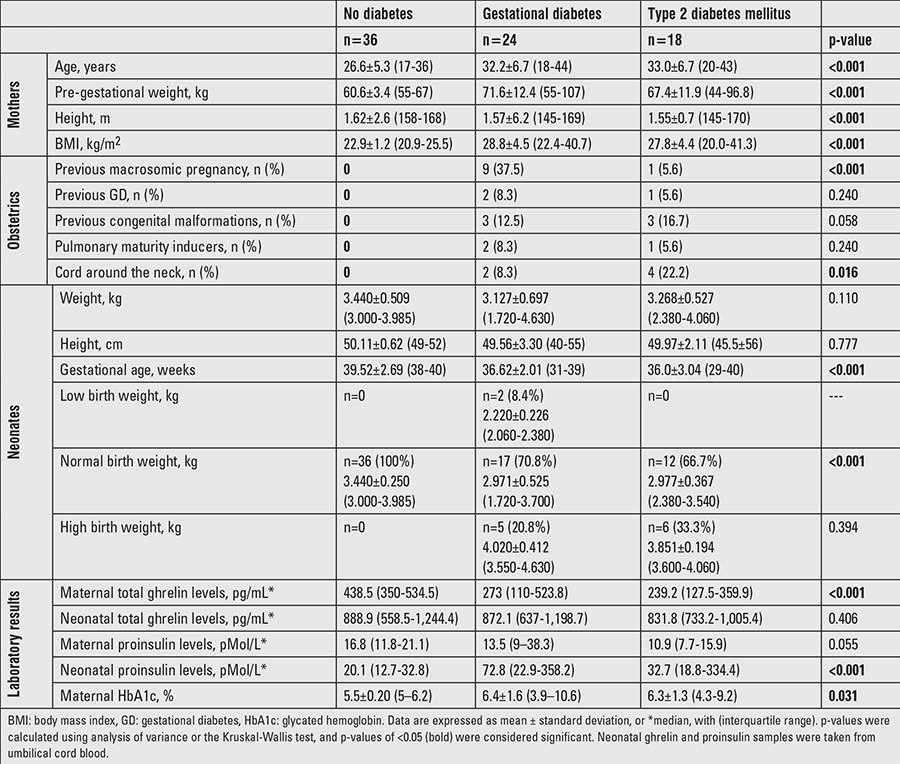
Characteristics of the study sample

**Table 2:**
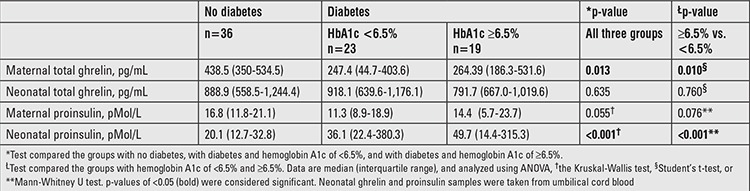
Total ghrelin and proinsulin levels in the mothers and infants according to glycated hemoglobin levels

**Table 3:**
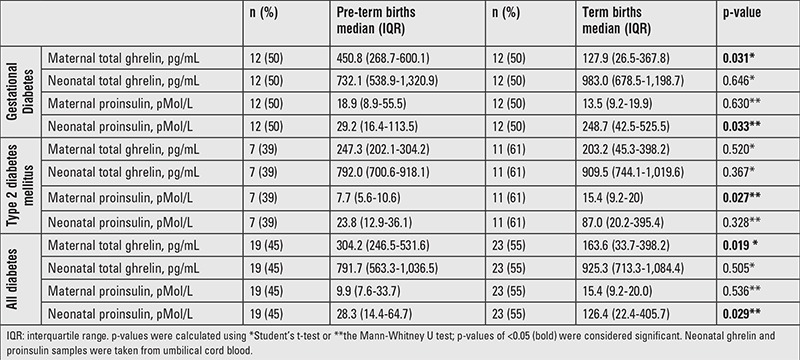
Total ghrelin and proinsulin levels among women with diabetes and their offspring in preterm and term birth
